# Clinical and dGEMRIC Evaluation of Microfragmented Adipose Tissue Versus Hyaluronic Acid in Inflammatory Phenotype of Knee Osteoarthritis: A Randomized Controlled Trial

**DOI:** 10.3390/biomedicines13092301

**Published:** 2025-09-19

**Authors:** Vilim Molnar, Željko Jeleč, Eduard Rod, Damir Hudetz, Petar Brlek, Igor Borić, Vid Matišić, Jana Mešić, Eduard Stjepan Pavelić, Dinko Vidović, Dejan Blažević, Fabijan Čukelj, Srećko Sabalić, Josip Štivičić, Tomislav Dujmović, Mario Starešinić, Martin Čemerin, David Glavaš Weinberger, Iva Molnar, Martina Smolić, Dragan Primorac

**Affiliations:** 1 St. Catherine Specialty Hospital, 10000 Zagreb, Croatia; zeljko.jelec@svkatarina.hr (Ž.J.); eduard.rod@svkatarina.hr (E.R.); ortohud@gmail.com (D.H.); petar.brlek@svkatarina.hr (P.B.); igor.boric@svkatarina.hr (I.B.); vid.matisic@svkatarina.hr (V.M.); jana.mesic@svkatarina.hr (J.M.); eduard.pavelic@svkatarina.hr (E.S.P.);; 2 Department of Physiotherapy, University North, 42000 Varaždin, Croatia; 3 Department of Orthopaedics and Traumatology, University Hospital Dubrava, 10000 Zagreb, Croatia; martincemerin@gmail.com; 4 School of Medicine, Josip Juraj Strossmayer University of Osijek, 31000 Osijek, Croatia; 5 Department of Molecular Biology, Faculty of Science, University of Zagreb, 10000 Zagreb, Croatia; 6 School of Medicine, University of Split, 21000 Split, Croatia; fabijancukelj@gmail.com (F.Č.); ssabalic@gmail.com (S.S.); 7 Department of Health Studies, University of Split, 21000 Split, Croatia; 8 Department of Traumatology, Sestre Milosrdnice University Hospital Center, 10000 Zagreb, Croatia; dinko.vidovic@gmail.com (D.V.); dejan.blazevic@live.com (D.B.); dawbgl11@gmail.com (D.G.W.); 9 School of Medicine, University of Zagreb, 10000 Zagreb, Croatia; mstaresinic@yahoo.com; 10 School of Dental Medicine, University of Zagreb, 10000 Zagreb, Croatia; 11 School of Medicine, Catholic University of Croatia, 10000 Zagreb, Croatia; 12 University of Applied Health Sciences, 10000 Zagreb, Croatia; 13 Department of Orthopaedics and Traumatology, University Hospital Split, 21000 Split, Croatia; 14 Clinic for Surgery, Department of General and Sports Traumatology, University Hospital Merkur, 10000 Zagreb, Croatia; josipstivicic@gmail.com (J.Š.);; 15 Health Centre Zagreb County, 10310 Ivanić Grad, Croatia; iva.mohler55@gmail.com; 16 Faculty of Dental Medicine and Health Osijek, Josip Juraj Strossmayer University of Osijek, 31000 Osijek, Croatia; 17 Forensic Science Program, Department of Biochemistry & Molecular Biology, The Pennsylvania State University, State College, PA 16802, USA; 18 The Henry C. Lee College of Criminal Justice and Forensic Sciences, University of New Haven, West Haven, CT 06516, USA; 19 Sana Kliniken Oberfranken, 96450 Coburg, Germany; 20 Medical School, University of Rijeka, 51000 Rijeka, Croatia; 21 Medical School, University of Mostar, 88000 Mostar, Bosnia and Herzegovina; 22 National Forensic Sciences University, Gandhinagar 382007, India

**Keywords:** adipose tissue, hyaluronic acid, injections, intra-articular, knee joint, mesenchymal stem cells, osteoarthritis

## Abstract

**Background**: Knee osteoarthritis (OA) is a leading cause of disability, with limited therapies that modify both symptoms and structural degeneration. Autologous microfragmented adipose tissue (MFAT) has emerged as a promising regenerative option, especially in phenotypically distinct OA subgroups. This randomized controlled trial evaluated the clinical and structural efficacy of intra-articular MFAT versus hyaluronic acid (HA) in patients with early to moderate inflammatory phenotype knee OA. **Methods**: Fifty-three patients were randomized in a 2:1 ratio to receive either MFAT (n = 35) or HA (n = 18). Patients were followed-up for six months post-injection and evaluated using patient-reported outcome measures (KOOS, WOMAC, VAS) and delayed gadolinium-enhanced MRI of cartilage (dGEMRIC). A responder analysis defined structural response as ≥10% increase in dGEMRIC in ≥3 of 7 predefined cartilage regions. **Results**: Both MFAT and HA led to statistically significant improvements in clinical scores and cartilage glycosaminoglycan content. MFAT showed greater mean improvements across most clinical and dGEMRIC measures, although without reaching statistical significance, except for KOOS Symptoms (MFAT: +25.0 vs. HA: +12.7, *p* = 0.008). Responder-level analysis revealed that all patients who demonstrated structural response also experienced clinically meaningful pain improvement (KOOS Pain ≥ 10), while no patient showed structural benefit without parallel symptomatic relief. **Conclusions**: MFAT led to greater improvement in symptoms related to joint stiffness, swelling, and crepitus compared to HA, reflecting its potential benefit in targeting the inflammatory features of knee OA. Importantly, HA also led to significant clinical and structural improvements, supporting its continued role as a standard-of-care comparator in knee OA management. Furthermore, the correlation between dGEMRIC and clinical response suggests its utility as a predictive biomarker of treatment success.

## 1. Introduction

Osteoarthritis (OA) is the most common chronic progressive musculoskeletal disorder affecting the synovial joints and primarily involves the weight-bearing joints, such as the hips and knees [1]. Knee OA is characterized by structural alterations in the articular cartilage and subchondral bone, as well as in the infrapatellar fat pad (Hoffa’s fat pad), synovium, ligaments, and periarticular muscles, suggesting that OA is a disease of the entire joint [2,3].

The worldwide prevalence of OA is significant, with more than 240 million people affected globally [4]. The burden of knee OA has increased dramatically in the last decades, and it is expected to keep growing not only because of obesity and the aging of the population, but also because of other unknown independent contributions [5]. OA represents a major burden not only for affected individuals but also for healthcare systems and society at large [6]. The prevalence of symptomatic knee OA is estimated to be 10% and 18% (men and women, respectively) in adults older than 60 years [4].

OA is a multifactorial disease influenced by numerous risk factors, including occupational stress, high-level sports activity, previous joint injuries, obesity, and sex-related differences [7]. A growing body of evidence highlights that these risk factors contribute not only to the onset but also to the clinical heterogeneity and progression of knee OA [8]. As a chronic, debilitating condition of pain and decreased functionality, conservative treatments such as lifestyle changes and analgesic therapy are frequently inadequate. For end-stage disease, total knee arthroplasty (TKA) remains the gold standard option, leading to a substantial improvement in quality of life once conservative treatment has failed. Yet, TKA does not target the primary pathogenesis of OA, and there are no known medications or biologics that have been shown to stop or reverse OA progression in the early stages of the disease [9]. The limitations of subjective clinical evaluation in knee OA highlight the importance of developing objective imaging and biomarker-based approaches to complement clinical assessment [10].

This treatment disparity has heightened interest in the pathogenesis, molecular features, and phenotypic heterogeneity of OA. In recent literature, evidence for different clinical phenotypes of knee OA clusters based on clinical, radiographic, biochemical, and patient-related variables has been provided [11,12,13,14]. In a landmark phenotype-based OA paper, Dell’Isola et al. proposed six such phenotypes: minimal joint disease, biomechanical axis deviation, chronic pain, inflammatory, metabolic syndrome-associated, and cartilage/bone metabolism phenotype [12]. Recent imaging-based analyses have similarly introduced structural phenotypes of OA, including inflammatory, meniscus-cartilage, subchondral bone, atrophic, and hypertrophic phenotypes, further underscoring the heterogeneity of the disease [8]. These stratifications offer the potential for phenotype-based, individualized treatment strategies.

The inflammatory phenotype is of particular interest for biologic interventions due to the presence of synovitis and joint effusion, which is quantified using the Magnetic Resonance Imaging Osteoarthritis Knee Score (MOAKS) with synovitis/effusion scores of 2 or greater [12,15]. Synovitis plays a central role in OA pathophysiology, being closely associated with both pain and disease progression [16]. This phenotype, which excludes patients with mechanical axis deformities or systemic comorbidities, could potentially benefit from anti-inflammatory therapy such as mesenchymal stem cell (MSC) treatment. MSCs derived from adipose tissue possess strong immunomodulatory and anti-inflammatory properties [17]. Studies suggest that adipose-derived MSCs have superior immunomodulatory potential compared to those derived from bone marrow [17].

As for more recent biological treatments, intra-articular injection of autologous microfragmented adipose tissue (MFAT), which contains stromal vascular fraction (SVF) and MSCs, has presented encouraging results, lowering pain and improving joint function [18,19,20,21]. Clinical outcomes after MFAT treatment have been previously demonstrated in improved KOOS (Knee Injury and Osteoarthritis Outcome Score) and WOMAC (Western Ontario and McMaster Universities Osteoarthritis Index) scores. Moreover, delayed Gadolinium-Enhanced Magnetic Resonance Imaging of Cartilage (dGEMRIC) showed that the GAG content of the cartilage generally remained stable or even increased after MFAT treatment, in contrast to the expected age-related decline. Hyaluronic acid (HA), one of the most commonly used intra-articular treatments for knee OA, has demonstrated the potential to stimulate proteoglycan synthesis in in vitro studies [22]. However, in vivo evidence of this anabolic effect, particularly when assessed using GAG synthesis markers such as dGEMRIC, remains limited.

Even though both MFAT and HA intra-articular injections are routinely used in clinical practice for the treatment of knee OA, to our knowledge, no randomized controlled trial (RCT) to date has explored and compared their effects using validated patient-reported outcome measures (PROMs) and compositional imaging biomarkers (dGEMRIC) in an early to moderate OA phenotyped population.

This study aims to evaluate and compare the clinical and cartilage structural effects of MFAT and HA in patients with the inflammatory phenotype of knee OA, using validated PROMs and dGEMRIC. We also examine whether structural improvement correlates with symptomatic relief, aiming to identify imaging markers predictive of therapeutic response.

## 2. Materials and Methods

### 2.1. Study Design

A prospective, interventional, randomized, double-blind clinical trial was conducted as part of the European research project (KK.01.2.1.02.0173). The participants were recruited from February 2020, and the last participant was enrolled on 31 May 2023. The trial was coordinated by St. Catherine Specialty Hospital (Zagreb, Croatia) in collaboration with the Department of Orthopedic and Sports Traumatology at University Hospital Merkur and the Clinic for Traumatology at Sestre milosrdnice University Hospital Center. Ethical approval was obtained from the Institutional Review Board of each participating institution (St. Catherine Specialty Hospital: UR 22/5-I; UH Merkur: UR BR-0311-2758; UHC Sestre milosrdnice: 251-29-11/3-22-02). The study was registered at the ISRCTN clinical study registry (ID: ISRCTN88966184).

Study participants were adults aged 30 to 75 years with primary knee OA who met strict inclusion/exclusion criteria and were classified as having an inflammatory OA phenotype based on patient history, clinical findings, comorbidities, and MRI evaluation. Patients who reported bilateral knee symptoms underwent evaluation of both knees. If both knees met the radiological inclusion criteria, the knee with the more severe symptoms was selected for treatment and analysis.

A total of 53 patients were enrolled and randomly assigned in a 2:1 ratio to receive either a single intra-articular injection of autologous MFAT containing MSCs (n = 35) or HA (n = 18). Patients were monitored for 6 months after the intervention, using both patient-reported outcome measures (PROMs) and MRI. PROMs (KOOS, WOMAC, and VAS) were collected at baseline, 1 month, and 6 months. dGEMRIC imaging was performed at baseline and 6 months. The primary outcomes included the change in PROMs and dGEMRIC index values over the defined time points. Secondary outcomes involved examining the correlation between changes in clinical scores and changes in the dGEMRIC index.

All data were anonymized and stored in a secure, password-protected database in compliance with GDPR and Good Clinical Practice (GCP) guidelines.

### 2.2. Inclusion and Exclusion Criteria

The study sample was carefully selected to ensure group homogeneity and reduce biological variability. Strict inclusion and exclusion criteria were used to select a group of patients representing the inflammatory phenotype of knee OA, as described by Dell’Isola et al. [11,12]. This phenotype is characterized by cartilage damage and joint effusion, without significant deviation of the mechanical axis, and systemic comorbidities such as diabetes, obesity, or depression. Patients exhibiting features of other OA phenotypes, like excessive knee valgus or varus, metabolic syndrome, or chronic pain, were excluded.

Joint effusion was assessed quantitatively using the MOAKS for synovitis/effusion. Only patients with a synovitis/effusion score of 2 or 3 were eligible for inclusion. This indicates the presence of convexity of the suprapatellar bursa or joint capsule distension on axial MRI slices [15]. Patients with scores below two were excluded, as these findings suggest minimal or no joint effusion. A single musculoskeletal radiologist performed MOAKS synovitis/effusion scoring before group allocation and was therefore not blinded.

The reason for choosing this phenotype is its responsiveness to biological therapies that target intra-articular inflammation. Unlike mechanically driven OA, the inflammatory phenotype is a clinical profile where treatments such as MFAT or HA can directly affect the underlying disease process and potentially relieve symptoms by reducing synovial inflammation.

Key exclusion criteria included systemic inflammatory or malignant diseases, post-traumatic OA, prior surgery on the affected knee, BMI over 30, diabetes, recent intra-articular injections (within 3 months), and any findings indicating biomechanical, metabolic, or chronic pain phenotypes. A detailed list of inclusion and exclusion criteria is available in Table 1.

### 2.3. Randomization and Blinding

Participants were randomized in a 2:1 ratio in favor of the MFAT group. This allocation was chosen to increase exposure to the novel treatment and to obtain more precise estimates of its effects, while maintaining HA as an active comparator. Randomization was performed for each patient at the time of enrollment using random.org. All participants underwent subcutaneous abdominal lipoaspiration and adipose tissue microfragmentation with the Lipogems^®^ system, regardless of treatment group, to maintain blinding. MFAT samples from patients in the HA group were cryopreserved for future use. All participants signed an informed consent form before enrollment and were assigned a unique, anonymized code for data tracking purposes. This was a double-blind study. Both patients and the radiologist performing dGEMRIC analysis were blinded to group allocation. Patient-reported outcome measures (PROMs) were completed independently by participants using anonymized codes.

### 2.4. Interventions

#### 2.4.1. Lipoaspiration and MFAT Preparation

Adipose tissue harvesting was performed under sterile conditions following a standardized lipoaspiration protocol. With the patient lying supine on the operating table, local anesthesia was administered using 2% lidocaine. A small skin incision (6–8 mm) was made at the donor site. Approximately 250 mL of tumescent solution, consisting of 40 mL of 2% lidocaine and one ampoule of adrenaline diluted in saline, was infiltrated into the subcutaneous fat to minimize bleeding and mechanical trauma.

Fat was harvested using a blunt cannula inserted through the incision, with gentle back-and-forth movements under negative pressure. The aspirated lipoaspirate was immediately processed with the Lipogems^®^ Ortho Kit (Lipogems International SpA, Milan, Italy), a closed, sterile system designed for mechanical microfragmentation and washing of adipose tissue without enzymatic digestion or expansion. A measure of 7 mL of the resulting MFAT was transferred into 10 mL syringes for intra-articular injection in the MFAT group. MFAT samples from patients in the HA group were cryopreserved in the tissue bank for potential future use. After harvesting, an elastic compression bandage was applied to the donor site to prevent the formation of a subcutaneous hematoma.

#### 2.4.2. Injection Procedure

The same injection protocol was used for both treatment groups. With the patient in a supine position and the knee fully extended, the femoral condyle was marked with a surgical pen to identify the injection site. After sterile skin preparation, a 21-gauge needle was inserted into the synovial space of the affected knee under real-time ultrasound guidance. Synovial fluid was aspirated before administering the treatment. In the MFAT group, 7 mL of processed MFAT was injected intra-articularly (Lipogems^®^). In contrast, in the HA group, 60 mg of high-molecular-weight HA (Hyalubrix 60^®^, Fidia Farmaceutici, Abano Terme, Italy) was delivered through the same needle. The procedure was performed without anesthesia or sedation, except for local analgesia.

Adverse events and procedure-related complications were monitored and documented throughout the study, including at each follow-up visit (1 and 6 months). Adverse events were defined as any unfavorable and unintended clinical sign, symptom, or disease that occurred temporally in association with the intervention, regardless of whether they were considered related to the treatment. Both local (e.g., injection site pain, swelling) and systemic events (e.g., fever, allergic reaction) were evaluated. Safety data were gathered by a clinician blinded to group assignment.

### 2.5. Clinical Outcome Measures

#### 2.5.1. Patient-Reported Outcome Measures (PROMs)

Patient-reported outcome measures included the VAS for pain assessment, the KOOS, and the WOMAC. These questionnaires were administered at baseline, as well as at 1 month and 6 months after the intervention. Participants, who were blinded to the assigned therapy, completed the questionnaires.

#### 2.5.2. Minimal Clinically Important Difference (MCID) and Ceiling Effect Adjustments

To complement continuous outcome analyses with clinically meaningful interpretation, responder status was determined based on established MCID thresholds: ≥10-point improvement for KOOS Pain, ≥15-point improvement for WOMAC Total, and >2-point improvement for VAS Movement [23,24,25,26,27,28,29]. Patients with baseline scores exceeding predefined ceiling thresholds were excluded from responder analyses to avoid ceiling effects: KOOS Pain > 85, WOMAC Total < 15, and VAS Movement ≤ 2. These cutoffs reflect the minimum level of impairment needed to allow for detectable and clinically meaningful improvement, as supported by previous literature for specific MCID values [23,24,25,26,27,28,29].

### 2.6. Imaging Assessment

Imaging assessments before enrollment included plain radiographs (anteroposterior, lateral, Rosenberg, axial patella), full-length weight-bearing panoramic images, and MRI of the target knee. Imaging was used to confirm specific inclusion and exclusion criteria (Table 1).

Quantitative MRI of the knee was performed using the dGEMRIC on a 3T Siemens MAGNETOM^®^ Lumina scanner (Siemens Healthineers, Erlangen, Germany). Patients received an intravenous injection of the gadolinium-based contrast agent (Gd-DTPA, Dotarem^®^, Guerbet, France) at a dose of 0.2 mmol/kg, followed by a 20 min walk to allow the contrast agent to diffuse into the joint. The relaxivity of the contrast agent was consistent across all patients because it was administered under identical conditions: contrast agent temperature, magnetic field strength, and concentration. The injection took less than 5 min. Imaging was performed 120 min after injection. The dGEMRIC images were analyzed by an experienced musculoskeletal radiologist using syngo MR XA31 MapIt software (Siemens, Erlangen, Germany).

The dGEMRIC protocol and ROI segmentation were adapted from previously validated methodologies used in cartilage imaging studies [21,30]. Seven predefined cartilage ROIs were identified, representing the thickest areas of articular cartilage: the medial and lateral femoral condyles, femoral trochlea, medial and lateral tibial condyles, and both patellar facets. T1 relaxation times were measured in these regions using T1-mapping sequences, with values recorded at baseline and six months after the intervention. All follow-up MRI scans were conducted within a window of 6 months ± 6 weeks post-injection. Articular facets lacking cartilage, where measuring the dGEMRIC index was not possible, were labeled as “0.” All MRI analyses were performed by a single musculoskeletal radiologist experienced in cartilage imaging, who was blinded to the treatment group assignment. All imaging was interpreted by the same radiologist to ensure consistency and minimize inter-observer variability, although intra-observer reproducibility was not formally assessed.

### 2.7. Statistical Analysis

Statistical analysis was performed using IBM SPSS Statistics for Windows, Version 26.0 (IBM Corp., Armonk, NY, USA). Intention-to-treat analysis was not performed. Data were analyzed according to the per-protocol principle, including only participants who received the assigned intervention and completed follow-up without significant protocol deviations (n = 52 for PROMs analysis, n = 51 for dGEMRIC analysis).

The Shapiro–Wilk test, suitable for the sample size, was used to assess normality. Based on the data distribution, either parametric or non-parametric tests were applied. For within-group comparisons over time, either the paired samples *t*-test or the Wilcoxon signed-rank test was employed, depending on the data distribution and sample size. The Mann–Whitney U test was employed for between-group comparisons. For small subgroups (N < 20), non-parametric tests were preferred, even if the Shapiro–Wilk test did not reject normality, to enhance robustness.

To compare categorical outcomes, the Chi-square test or Fisher’s exact test was applied. A cross-tabulation analysis using Fisher’s exact test was also conducted to assess the association between structural (dGEMRIC-based) and clinical (KOOS Pain) response.

As part of the prespecified imaging objectives, we compared the proportion of ROIs demonstrating a clinically relevant improvement in dGEMRIC index (≥10%) between treatment groups. This responder-type analysis was defined before data unblinding and was based on previously reported thresholds for meaningful change in cartilage GAG content.

Clinical responder analysis was also conducted using the KOOS Pain subscale. A responder was defined as a patient who showed an improvement of 10 points or more from baseline to 6 months. To address potential ceiling effects, a sensitivity analysis was performed that excluded patients with a baseline KOOS Pain score of 85 or higher. To evaluate the impact of administered therapy, MCID thresholds were also presented for two other questionnaires: a WOMAC Total score of 15 points or more from baseline and a VAS in movement of 3 points or more from baseline.

To facilitate patient-level interpretation of imaging outcomes and enable direct comparison with clinical response, a composite dGEMRIC response criterion was defined. Patients were classified as dGEMRIC responders if three or more of the seven evaluated cartilage regions exhibited a ≥10% increase in dGEMRIC index at 6 months compared to baseline. This threshold was selected to reflect a generalized, rather than focal, structural response, accounting for biological variability and measurement noise at the single-ROI level. The criterion of ≥3 responsive regions was pragmatically chosen to ensure clinical interpretability while minimizing false-positive classification due to incidental fluctuations.

A *p*-value less than 0.05 was considered statistically significant. Graphs were generated using GraphPad Prism version 9.4.1 for Windows (GraphPad Software, San Diego, CA, USA). An a priori power analysis based on expected effect sizes in KOOS Pain and dGEMRIC changes indicated that the sample size would provide 70–80% power to detect significant differences between groups (*p* = 0.05).

## 3. Results

### 3.1. Participant Characteristics

**Figure 1:**
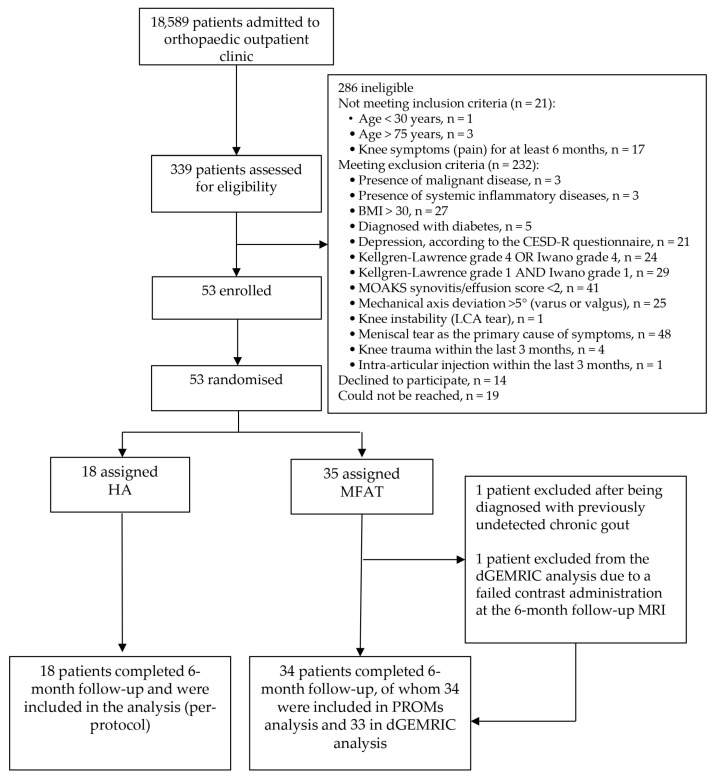
CONSORT flow diagram illustrating participant recruitment, eligibility screening, randomization, follow-up, and analysis. Of 339 patients assessed for eligibility, 53 were randomized to receive either microfragmented adipose tissue (MFAT) or hyaluronic acid (HA) injections. Eligibility was determined using strict radiographic, clinical, and MRI-based phenotyping. PROMs and dGEMRIC analyses were conducted according to the per-protocol principle. Reasons for exclusion are detailed, including specific inclusion and exclusion criteria, as well as technical issues affecting imaging analysis.

A total of 18,589 patients were screened in the orthopedic outpatient clinic, of whom 339 were assessed in detail for eligibility. Fifty-three patients with knee OA fulfilled the inclusion criteria and were included in the study. They were randomized into two treatment groups: 35 patients received autologous MFAT and 18 received HA. A detailed CONSORT flow diagram illustrating patient screening, randomization, follow-up, and analysis is shown in Figure 1. The mean age of the patients was 55.6 ± 9.4 years, and the overall cohort included 40 (75.5%) females and 13 (24.5%) males. No significant differences were observed in age or sex distribution between the groups (Table 2).

One patient from the MFAT group was excluded from all posttreatment analyses after being diagnosed with previously undetected chronic gout, which was retrospectively identified as the primary source of joint symptoms and rendered follow-up assessments invalid. Additionally, one female participant was excluded from the dGEMRIC analysis due to a failed contrast administration at the 6-month follow-up MRI.

### 3.2. Clinical Outcomes (PROMs)

There were no statistically significant baseline differences between the MFAT and HA groups in any of the assessed patient-reported outcomes, including KOOS subscales, WOMAC (total and subscale scores), or VAS (rest and activity scores). This confirms group comparability at enrollment and provides a robust foundation for evaluating differential treatment effects.

#### 3.2.1. KOOS

Both treatment groups showed statistically significant improvements across all KOOS subscales (Symptoms, Pain, Activities of Daily Living (ADL), Sport and Recreation (Sport/Rec), and Quality of Life (QoL)) at 1 and 6 months compared to baseline (*p* < 0.01 for all comparisons). In the MFAT group, these improvements were consistent and progressive, with all subscales showing statistically significant gains not only from baseline to 1 month but also from 1 to 6 months (*p* < 0.01), indicating a sustained therapeutic benefit over time.

In the HA group, all subscales showed significant improvement from baseline to both follow-ups. However, the magnitude of additional improvement between 1 and 6 months was smaller. It did not reach statistical significance for the KOOS subscales (*p* > 0.05, Wilcoxon signed-rank test), except for the KOOS QoL subscale (*p* = 0.028, Wilcoxon signed-rank test), suggesting a plateau in clinical benefit after the initial response. Full descriptive statistics for each KOOS subscale at all three timepoints (baseline, 1 month, and 6 months), including within-group comparisons and *p*-values, are provided in Supplementary Table S1.

**Figure 2:**
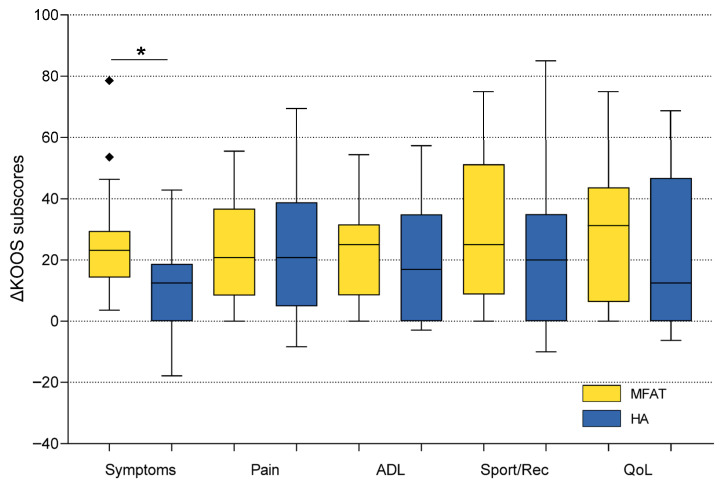
Comparison of KOOS subscale improvements (baseline to 6 months) between microfragmented adipose tissue (MFAT) and hyaluronic acid (HA) groups. Box plots illustrate the distribution of score changes, with median, interquartile range, and outliers. The MFAT group showed a significantly greater improvement in KOOS Symptoms (*p* = 0.008), while no significant between-group differences were observed in the other subscales. The Mann–Whitney U test was used for statistical analysis. Squares represent outliers. An asterisk indicates a statistically significant difference between groups. KOOS—Knee Injury and Osteoarthritis Outcome Score; ADL—Activities of Daily Living; Sport/Rec—Sports and Recreational Activities; QoL—Quality of Life.

The mean changes (baseline to 6 months) for the MFAT group were as follows: KOOS Pain 23.0 ± 15.0, Symptoms 25.0 ± 15.6, ADL 22.6 ± 14.8, Sport/Rec 29.7 ± 24.6, and QoL 29.8 ± 22.0. Corresponding values for the HA group were: Pain 22.4 ± 22.0, Symptoms 12.7 ± 16.0, ADL 19.3 ± 19.6, Sport/Rec 19.7 ± 23.7, and QoL 23.6 ± 25.4. Between-group comparisons of KOOS subscale improvements (baseline to 6 months) revealed no statistically significant differences for Pain, ADL, Sport/Rec, or QoL (all *p* > 0.05, Mann–Whitney U test). However, a significant difference was observed in the KOOS Symptoms subscale, with the MFAT group demonstrating greater improvement than the HA group (mean change: 25.0 vs. 12.7; U = 168.5; *p* = 0.008), reflecting a superior mid-term effect of MFAT on joint-related symptoms such as stiffness, swelling, and crepitus (Figure 2, Table 3).

#### 3.2.2. WOMAC

Both treatment groups showed significant improvements in all WOMAC subscale scores (Pain, Stiffness, Function, and Total score) at 1 and 6 months compared to baseline (for all within-group comparisons, *p* < 0.01, Wilcoxon signed-rank test).

Improvement in the MFAT group was incremental and broad-based, with all subscales demonstrating statistically significant improvement not only from baseline at 1 month but also from 1 to 6 months (*p* < 0.01), suggesting therapeutic benefits that persisted over time.

**Figure 3:**
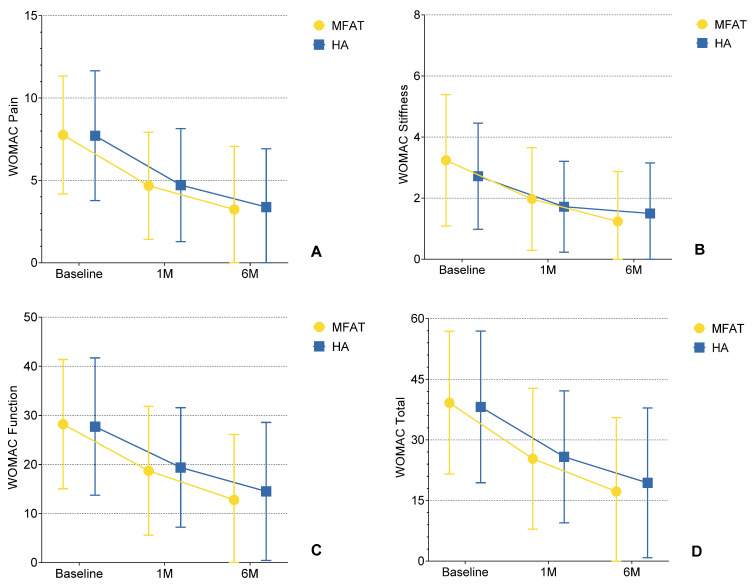
(**A**–**D**) Changes in WOMAC subscale scores (Pain, Stiffness, Physical Function, and Total) over time following intra-articular injection of microfragmented adipose tissue (MFAT) or hyaluronic acid (HA). Each plot depicts mean values ± standard deviation (SD) error bars at baseline, 1-month, and 6-month follow-up. During the follow-up period from 1 to 6 months, all outcomes in all subscales were statistically significant in comparison to baseline (*p* < 0.01), demonstrating a sustained treatment effect in the MFAT group. In contrast, significant improvements from baseline to 1 month (*p* < 0.01) were observed in the HA group, followed by no significant change from 1 to 6 months (*p* > 0.05), suggesting a plateau effect between 1 and 6 months. These findings visually support the hypothesis that MFAT provides more prolonged clinical improvement in inflammatory knee OA compared to HA.

The HA group gained a significant benefit in all domains from baseline to both follow-up evaluations (*p* < 0.01). However, the magnitude of further improvement from 1 to 6 months was smaller and not statistically significant for any of the subscales (*p* > 0.05), suggesting that much of the clinical benefit was realized early with limited additional improvement after that time. The longitudinal trends in each subscale are illustrated in Figure 3A–D.

Descriptive statistics (mean ± SD) for all WOMAC subscales at baseline, 1 month, and 6 months in both treatment groups, as well as within-group *p*-values for each time interval, are provided in Supplementary Table S2.

There were no statistically significant differences between the groups based on the improvement (delta) in WOMAC Pain, Stiffness, Function, or Total scores from baseline to 6 months (all *p* > 0.05, Mann–Whitney U test). However, mean improvements were consistently larger for the MFAT group on all subscales (Supplementary Table S3).

In conclusion, both MFAT and HA provided clinically significant benefits, with similar efficacy between the two groups, as indicated by the WOMAC score.

#### 3.2.3. Visual Analog Scale (VAS)

The two treatment groups experienced statistically significant reductions in pain (as measured by the VAS in resting and during motion) at both 1 and 6 months compared to baseline (*p* < 0.01 for most comparisons within groups).

In the MFAT group, the pain-relieving effect was sustained beyond 1 month, with the reduction in resting and movement pain remaining statistically significant from 1 to 6 months (*p* < 0.01), indicating progressive and durable therapeutic effects.

In the HA group, pain was also significantly reduced between baseline and both follow-up moments. After the first month, the decrease in movement pain stabilized, and there was no further statistically significant improvement between 1 and 6 months (*p* = 0.115). This indicates a reduced mid-term analgesia for HA in dynamic conditions. In contrast, resting pain continued to decline significantly between 1 and 6 months (*p* = 0.023), suggesting a more durable effect on static pain. Full descriptive statistics for both VAS conditions, including mean ± SD values and within-group *p*-values, are provided in Supplementary Table S4.

No statistically significant differences were found between the two groups in delta scores (baseline to 6 months) for resting and movement pain, comparing the delta scores from baseline to 6 months (*p* > 0.05). This indicates that both approaches yield comparable long-term analgesic effects, as measured by the VAS scale.

#### 3.2.4. Responder Analysis

In addition to analyses of continuous outcomes, the proportions of responders in each treatment group were calculated using previously established minimal clinically important difference (MCID) thresholds: KOOS Pain (≥10), WOMAC Total (≥15), and VAS Movement (>2). Patients with baseline ceiling effect (KOOS Pain > 85, WOMAC Total < 15, VAS Movement ≤ 2) were removed from these analyses.

**Figure 4:**
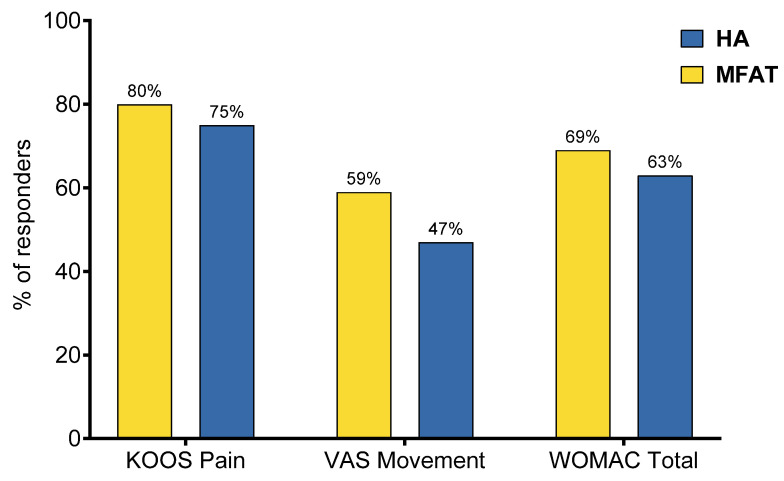
Proportion of responders in each treatment group according to established minimal clinically important difference (MCID) thresholds: KOOS Pain (≥10), VAS Movement (>2), and WOMAC Total (≥15). Despite no statistically significant differences (*p* > 0.05 for all comparisons, Fisher’s exact test for KOOS Pain and WOMAC Total, Chi-square test for VAS Movement), the MFAT group consistently showed higher responder rates across all outcome measures, suggesting a trend toward greater clinical benefit.

Despite none of the between-group comparisons being statistically significant, the MFAT group had constantly higher response rates in all observed outcomes: KOOS Pain (80% MFAT vs. 75% HA, *p* = 0.717, Fisher’s exact test), VAS Movement (59% MFAT vs. 47% HA, *p* = 0.452, Chi-square test) and WOMAC Total (69% MFAT vs. 63% HA, *p* = 0.741, Fisher’s exact test) (Figure 4). Exact numbers and percentages of responders and non-responders for each outcome are provided in Supplementary Table S5.

These responder-based analyses provide an additional level of clinical interpretation, indicating that a greater percentage of MFAT-treated patients experienced clinically meaningful changes, despite the group-level differences not reaching statistical significance.

Collectively, these findings suggest that although significant and clinically relevant improvements for knee OA symptoms are associated with both MFAT and HA, MFAT might be associated with a more persistent and gradual benefit over time, particularly for symptoms related to joint stiffness and effusion.

### 3.3. Imaging Outcomes (dGEMRIC)

To demonstrate the therapeutic efficacy of intra-articular treatments on cartilage matrix composition, dGEMRIC was employed as a well-established MRI biomarker of GAG content. This provides an indirect estimation of GAG content by using T1 after contrast administration, which can be considered a measure of the biochemical state.

A total of 357 cartilage regions of interest (ROIs) were assessed using the dGEMRIC technique at baseline and 6 months following treatment. Five ROIs were excluded from analysis because their baseline dGEMRIC index value was zero, which prevented the calculation of percentage change. Accordingly, 352 ROIs were available for the ultimate comparison analysis.

**Figure 5:**
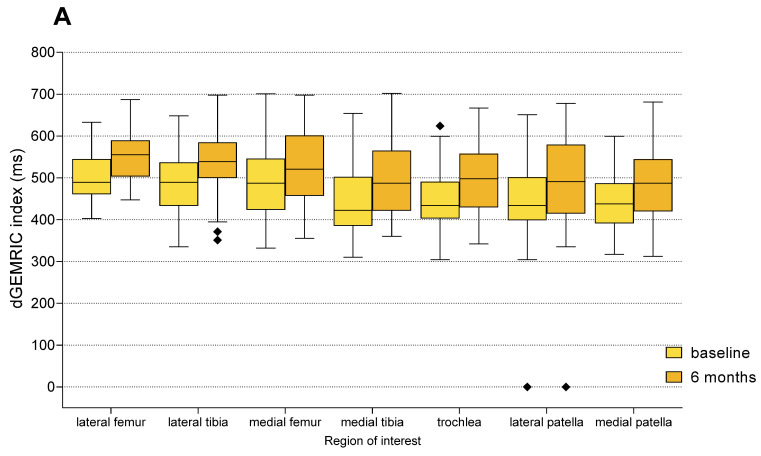

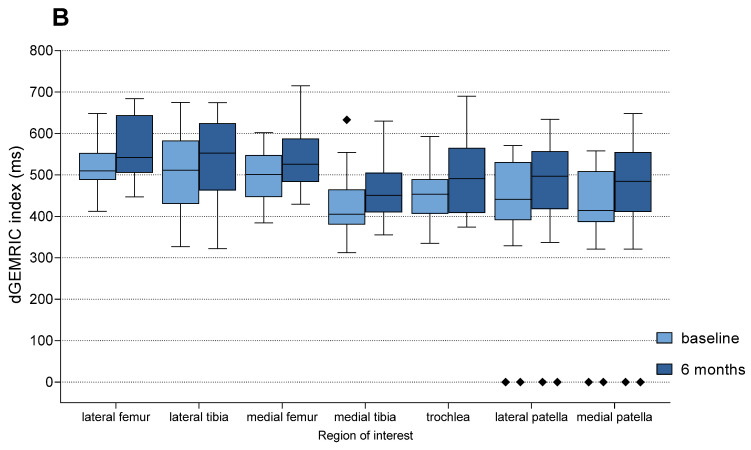
Changes in dGEMRIC indices from baseline to 6 months follow-up in each anatomical region of interest (ROI), shown separately for the MFAT (**A**) and HA (**B**) treatment groups. Boxplots represent median and interquartile range, with light colors indicating baseline values and darker shades representing follow-up measurements. Statistically significant improvements were observed in all compartments within both groups (Paired *t*-test or Wilcoxon signed-rank test, *p* < 0.01 for all regions). Squares represent outliers.

Intra-group analysis with the use of Paired *t*-test (parametric data) or Wilcoxon signed-rank test (non-parametric delta values; dGEMRIC for the medial tibia and sample size < 20) revealed statistically significant increases in dGEMRIC index from baseline to 6 months in all seven anatomical regions in both treatment groups (*p* < 0.01 for all comparisons) (Figure 5, Supplementary Table S6). These results indicate that treatment with both MFAT and HA resulted in a detectable increase in GAG content in articular cartilage during the 6-month follow-up period.

A clinically relevant improvement, defined as a ≥10% increase in dGEMRIC index compared to baseline, was observed in 171 of 352 ROIs (48.58%). Non-significant changes (<10% increase or decrease) were observed in 180 ROIs (51.14%), while only one ROI (0.28%) demonstrated a clinically significant deterioration (≥10% decrease). The proportion of ROIs with clinically relevant improvement was 45.08% (55/122) in the HA group and 50.43% (116/230) in the MFAT group. The only clinically significant deterioration was observed in the MFAT group (1/230 ROIs; 0.43%), while no deteriorations were recorded in the HA group.

**Figure 6:**
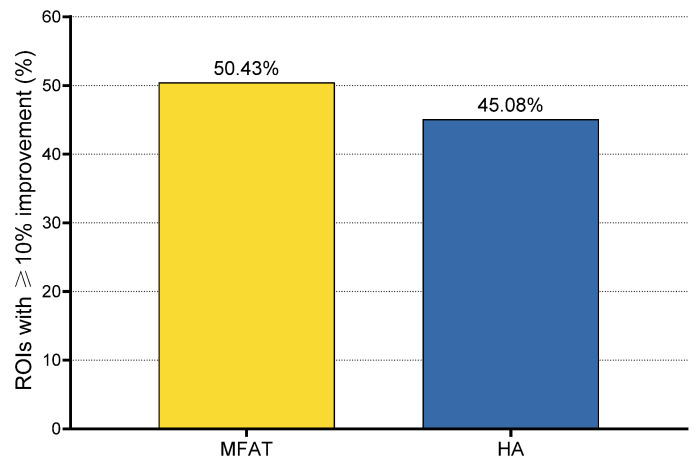
Proportion of regions of interest (ROIs) showing clinically relevant improvement (≥10% increase in dGEMRIC index) 6 months after treatment. Clinically relevant improvement was observed in 55 of 122 ROIs (45.08%) in the HA group and 116 of 230 ROIs (50.43%) in the MFAT group. One clinically relevant deterioration (≥10% decrease) was observed in the MFAT group (0.43%), while no deteriorations were recorded in the HA group.

Although the proportion of ROIs with clinically meaningful improvement was higher for the MFAT group, the distribution of outcome categories did not differ significantly between the two groups (χ^2^ = 1.52, df = 2, *p* = 0.467). The rates of improvement between the groups are compared in Figure 6.

Posttreatment percentage change in the dGEMRIC index was compared between the MFAT and HA groups for each ROI. Although no statistically significant differences were detected (all *p* > 0.05, Mann–Whitney U test), ROIs in the MFAT group consistently showed greater mean improvements in GAG content compared to the HA group. The most notable mean change in the dGEMRIC index was observed in the medial tibia (+13.3% MFAT vs. +9.2% HA). Additionally, every ROI in the MFAT group, except the medial femur, demonstrated a mean improvement greater than the established MCID of 10%. In contrast, none of the ROIs in the HA group achieved such enhancements. The regional distribution of baseline indices and treatment responses is shown in Table 4.

To put these results into a patient perspective, we also defined dGEMRIC responders as patients in whom at least three out of seven ROIs demonstrated a clinically relevant improvement (≥10% increase in dGEMRIC index). Evaluating by this cutoff point, 50% (9/18) of the HA-group patients and 54.5% (18/33) of the MFAT-group patients were evaluated as dGEMRIC responders. But there was no significant difference in responder rate between the two groups (χ^2^ = 0.001, *p* = 0.986).

### 3.4. Correlation Between Clinical and Imaging Outcomes

A responder-level analysis was conducted to evaluate the link between structural and symptomatic improvements after treatment. Patients were classified as dGEMRIC responders if at least three out of seven cartilage regions assessed showed a minimum 10% increase in dGEMRIC index from baseline to six months. KOOS Pain responders were defined as those with a 10-point or greater improvement from baseline, aligning with the established MCID.

One patient was excluded from this analysis due to missing dGEMRIC data at follow-up, and an additional six patients were excluded because their baseline KOOS Pain score was 85 or higher, indicating the presence of a ceiling effect and making response classification impossible.

Among the remaining 45 patients, a statistically significant association was found between dGEMRIC and KOOS Pain responder status (*p* = 0.001, Fisher’s exact test). All dGEMRIC responders (n = 23) were KOOS Pain responders, and none of the KOOS Pain non-responders (n = 8) were dGEMRIC responders. It is noteworthy that the positive predictive value (PPV) of dGEMRIC response for KOOS Pain improvement was 100%, indicating that where structural improvement was observed, symptomatic relief consistently followed (Table 5).

Similar trends were observed in the subgroup analyses for both arms. The association remained statistically significant in the MFAT group (*p* = 0.042, Fisher’s exact test), but did not reach statistical significance in the HA group (*p* = 0.077, Fisher’s exact test).

There was no statistical correlation between the dGEMRIC response and improvement in KOOS Symptoms for the total population or when divided by treatment (all *p* > 0.05). As the KOOS Symptoms subscale is broad and multifaceted, only the KOOS Pain subscale was retained for further analysis and interpretation.

## 4. Discussion

This randomized controlled trial, to our knowledge, is the first to directly compare MFAT and HA in a carefully selected group of patients with early to moderate inflammatory phenotype of knee OA. By combining validated clinical scores with advanced cartilage imaging, we aimed to gain a more complete picture of treatment response. Although MFAT has been tested against other injectables like PRP [31], there has been a surprising lack of head-to-head data versus the most widely used standard-of-care injection, such as HA. Our findings help to fill that gap and offer new insights into how biologic therapies like MFAT may perform in this particular OA phenotype.

In this RCT, we assessed the clinical effectiveness of MFAT versus HA in patients with knee OA, utilizing validated PROMs, specifically KOOS, WOMAC, and VAS. These tools are widely recognized for capturing a range of symptom domains, including pain intensity, joint stiffness, functional limitations, and quality of life. Participants were evaluated at baseline, 1 month, and 6 month follow-ups, ensuring temporal sensitivity to detect short-to-mid-term therapeutic effects.

Both treatments resulted in meaningful clinical improvements at both 1- and 6-month follow-ups compared to baseline. However, MFAT exhibited more durable effects across multiple PROMs, particularly between 1 and 6 months, suggesting a progressive therapeutic trajectory. In contrast, the HA group showed early benefits that largely plateaued beyond the first month on most PROMs subscales. These dynamics may be particularly relevant for physicians seeking longer-lasting, non-surgical solutions for managing early to moderate knee OA.

Between-group comparisons at 6 months revealed no statistically significant differences in KOOS Pain, ADL, Sport/Rec, or QoL domains. However, the MFAT group demonstrated significantly greater improvement in KOOS Symptoms compared to HA (mean change 25.0 vs. 12.7; *p* = 0.008). This particular subscore reflects the symptomatology of joint stiffness, swelling, and mechanical symptoms, which are primarily influenced by local inflammation and synovial activity. The better MFAT performance in this subscale may indicate that its mechanism of action in the joint is not purely mechanical supplementation, as seen with HA, but rather a more significant immunomodulatory impact on joint homeostasis. This is further supported by dGEMRIC results, which show a greater increase in GAG content in the MFAT group, consistent with its proposed regenerative and anti-inflammatory properties. These findings align with previous preclinical and early clinical studies indicating that adipose-derived MSCs can modulate inflammation and support tissue repair, which is likely to explain their clinical benefit in inflammatory OA [17,32]. However, given the lack of investigation into the relative impact of these two treatments on cartilage biochemistry, especially with such a sensitive structural biomarker as dGEMRIC, clinicians must admit their lack of understanding of the true disease-modifying potential of both.

The results were mirrored in the WOMAC and VAS outcomes. The MFAT group exhibited consistent and statistically significant improvements in all WOMAC domains between the two follow-up points (*p* < 0.01). In contrast, the HA group showed a significant increase from baseline to 1 month but no further improvements by 6 months (*p* > 0.05). A similar tendency was observed in VAS scores, particularly in the case of movement-related pain, which plateaued at 1 month in the HA group but continued to decrease significantly in the MFAT group, highlighting the sustained analgesic potential of MFAT.

To additionally place the clinical impact in context, we conducted responder analysis based on established MCID thresholds: ≥10 for KOOS Pain, ≥15 for WOMAC Total, and ≥3 for VAS Movement. While none of the between-group differences reached statistical significance, the MFAT consistently demonstrated higher responder rates: KOOS Pain (80% vs. 75%), WOMAC Total (69% vs. 63%), and VAS Movement (59% vs. 47%). Although the differences were not statistically significant, the trend favors MFAT, with more patients experiencing meaningful clinical improvement.

The choice of a 10-point MCID for KOOS Pain is well supported in the literature. Several anchor- and distribution-based studies in knee OA populations have identified different thresholds as clinically meaningful for this subscale, depending on the clinical context and method of estimation, which confirms that our choice of MCID for the KOOS pain scale was correct [23,24,25,26]. In this light, the mean KOOS Pain improvement in both treatment arms exceeded the MCID threshold, reinforcing the clinical utility of both interventions.

Although no prior studies have directly compared MFAT and HA, previous RCTs have compared HA with other biologic therapies, including SVF and culture-expanded MSCs. Hong et al. reported superior outcomes for SVF over HA at 6 months, including improvements in VAS and WOMAC scores, while a recent meta-analysis by Han et al., comparing SVF and HA, also favored cellular therapy in terms of pain reduction and functional improvement [33,34]. Similarly, meta-analysis of expanded MSCs suggest superior efficacy over HA, especially in early pain relief and functional improvement [35]. However, these studies differ in tissue source, processing methods (e.g., enzymatic digestion, culture expansion), and often lack phenotype stratification. Our study contributes high-quality randomized evidence supporting the clinical benefit of non-enzymatically processed, autologous MFAT, applied as a single intra-articular injection, in a phenotypically homogeneous cohort. Finally, no side effects were observed in any of the groups during the follow-up period, supporting the excellent safety and tolerability profile of both treatments within the studied timeframe.

DGEMRIC is a validated quantitative imaging biomarker that reflects GAG content within the cartilage extracellular matrix. The technique relies on the inverse relationship between the negatively charged gadolinium contrast agent and GAG molecules, providing an indirect but precise measurement of cartilage biochemical integrity and GAG distribution [30]. Its use in clinical research enables objective, non-invasive assessment of cartilage biochemical quality and has been increasingly adopted as a surrogate marker for early cartilage degeneration and treatment response. To our knowledge, this study represents the first RCT to evaluate the biological effects of MFAT compared to HA using dGEMRIC in patients with knee OA.

In our cohort, both MFAT and HA demonstrated statistically significant within-group increases in dGEMRIC indices across all seven evaluated anatomical compartments, suggesting measurable improvement in cartilage GAG content over the six-month follow-up period. A key methodological distinction of this study is the adoption of a ≥10% increase in the dGEMRIC index as the threshold for clinically relevant improvement. This deviates from prior studies conducted at St. Catherine Specialty Hospital, which used a ≥15% cutoff when evaluating the biological effects of MFAT on cartilage quality [19,21]. Given recent advances in the reproducibility of dGEMRIC imaging and validation from external studies, a 10% threshold was deemed appropriate for detecting meaningful biochemical changes in cartilage [36,37,38]. The use of this lower threshold is therefore justified both analytically and by emerging evidence from the broader field.

When stratified by this criterion, 50.4% of cartilage regions treated with MFAT and 45.1% of those treated with HA exhibited a clinically relevant improvement, with only one ROI (in the MFAT group) showing a clinically significant deterioration. Although no statistically significant difference was found between the groups in the proportion of ROIs with clinically relevant improvement, this analysis was part of the prespecified primary imaging outcome, aligning with the trial’s objective to integrate quantitative imaging with clinical evaluation in a phenotype-specific OA population. The nearly identical proportion of imaging responders in both treatment arms (54.5% MFAT vs. 50% HA) further supports the conclusion that both therapies led to measurable improvements in cartilage composition, but with comparable overall efficacy (χ^2^ = 0.001, *p* = 0.986).

Our findings indicate that both MFAT and HA treatments contribute to increased cartilage GAG content, as evidenced by higher dGEMRIC index values across all evaluated regions. While no statistically significant differences were observed between the two therapies at the selected threshold, the observed improvements likely arise from different underlying mechanisms. MFAT may exert its effect primarily through adipose-derived MSCs that promote anabolic repair and extracellular matrix synthesis, whereas HA likely acts by modulating chondrocyte metabolism and stimulating GAG production [17,22,39,40]. Recent evidence further supports these mechanistic distinctions. Adipose-derived MSCs exert their effects largely through immunomodulation of the synovial microenvironment, mediated by paracrine signaling, extracellular vesicles, and suppression of catabolic mediators [17,41]. MFAT therefore provides not only a cellular reservoir but also trophic support that may enhance joint homeostasis and cartilage repair [41]. Conversely, intra-articular HA has been shown to exert additional biochemical effects beyond lubrication, including modulation of pro-inflammatory cytokines, antioxidant activity, and stimulation of proteoglycan and collagen synthesis via CD44-mediated signaling [42]. Together, these findings underscore that MFAT and HA operate through distinct but complementary mechanisms, which may explain the differences in clinical and structural outcomes observed in our trial. The sensitivity of the dGEMRIC technique in capturing these subtle biochemical changes highlights its potential utility in the longitudinal monitoring of intra-articular therapies aimed at modifying OA progression. The GAG increases observed in both groups suggest a possible structure-modifying effect, particularly relevant for early intervention. However, longer-term studies are needed to determine whether these biochemical improvements translate into true disease modification.

Interestingly, these findings contrast with the only other study to assess the impact of HA on cartilage GAG content via dGEMRIC. In a non-randomized clinical trial by van Tiel et al., three weekly injections of intra-articular HA in patients with early-stage OA resulted in no significant modification of the dGEMRIC index after 14 weeks, despite clinically improved symptoms [37]. Several key differences may explain the divergent outcomes, including variations in HA formulation, injection regimen, patient selection, and notably, follow-up duration (14 weeks vs. 6 months). Unlike van Tiel’s cohort, our study focused on a phenotypically stratified population and employed a longer observation period. These findings suggest that, under specific conditions, HA may indeed induce modest but measurable improvements in cartilage composition over time, an effect that may have been overlooked in previous studies with shorter follow-up and less targeted patient selection.

A comparison with previous studies conducted at St. Catherine Specialty Hospital also emphasizes the importance of patient selection and disease severity. In contrast to the current cohort of patients with KL tibiofemoral or Iwano patellofemoral OA grade 2–3, previous studies have exclusively included patients with end-stage OA (KL 4) [19,21]. In our research, the tendency for a dGEMRIC response in patients with less severe OA provides support for the hypothesis that the earlier the intervention, the higher the corresponding biological gain, particularly in the cartilage matrix composition. Meng et al. also reported alterations in GAG content following MSC treatment, which underscores the role of biologics in modifying cartilage biochemistry [39]. The findings of these studies demonstrate the potential for MFAT to provide a clinically relevant impact on cartilage tissue quality as well as symptomatic relief, as indicated by patient-reported outcome measures in inflammatory knee OA patients.

On the other hand, no published study has utilized dGEMRIC or directly compared MFAT and HA, underscoring the novelty of our combined structural and symptomatic assessment. Likewise, HA has been widely studied and is known to temporarily improve joint lubrication and modulate inflammation. However, the lack of studies exploring their comparative effects on cartilage biochemistry, particularly with sensitive structural biomarkers like dGEMRIC, limits the clinical understanding of their potential disease-modifying properties. Whether such biochemical improvements, especially after HA treatment, represent sustained remodeling or transient changes remains unclear and warrants longer-term follow-up.

A key objective of this study was to evaluate whether MRI-based improvements in cartilage composition, as reflected by the dGEMRIC index, are aligned with pain relief. To assess the relationship between structural and symptomatic response on an individual-patient level, we performed a responder-level analysis. Rather than relying on mean dGEMRIC values across all cartilage regions, which may mask focal improvements, we defined structural response based on the number of regions showing clinically meaningful improvement. This approach avoids bias from isolated worsening events and better reflects the true biological response of selective joint lesions. No relationship was observed between dGEMRIC response and KOOS Symptom improvement, likely due to the more subjective and variable nature of the Symptoms’ subscale, which includes stiffness, grinding, and joint awareness, parameters that may be less directly associated with the biochemical composition of cartilage. The KOOS Pain scale, in contrast, is more closely tied to nociceptive burden, making it a more suitable comparator for imaging biomarkers such as dGEMRIC [43].

Our results show a strong correlation between the dGEMRIC-defined structural response and KOOS Pain improvement. Strikingly, all patients classified as dGEMRIC responders also exceeded the responder threshold on the KOOS Pain scale, both in the pooled cohort and within each treatment arm, yielding a positive predictive value (PPV) of 100%. To our knowledge, this is the first study to demonstrate such a high predictive concordance between an imaging biomarker and a clinically meaningful patient-reported outcome in knee OA. Conversely, clinical improvement alone was not always accompanied by measurable changes on dGEMRIC. Approximately one-third of KOOS Pain responders were not classified as dGEMRIC responders, underscoring that symptomatic response may arise through additional pathways, including anti-inflammatory effects, placebo response, and others. Importantly, none of the KOOS Pain non-responders exhibited a structural response as measured by dGEMRIC, suggesting that meaningful biological remodeling is unlikely in the absence of clinical benefit. This consistent alignment between structural and symptomatic response reinforces the translational value of dGEMRIC as a clinically meaningful biomarker for biologic interventions in OA. Collectively, our findings strengthen the rationale for integrating imaging endpoints such as dGEMRIC into future OA trials, particularly those evaluating biologic therapies aimed at achieving both structural modification and symptomatic improvement.

Previous dGEMRIC-based studies in osteoarthritis have predominantly focused on advanced disease stages or evaluated cartilage repair following surgical interventions [19,21,44]. Very few have systematically investigated changes in GAG content in response to biological therapies such as MFAT or HA. By demonstrating measurable increases in GAG content in KL tibiofemoral or Iwano patellofemoral OA grade 2–3 patients following both MFAT and HA treatment, our study provides a foundation for designing future research that could safely explore biologic interventions in more advanced disease stages, including KL 4, using sensitive imaging endpoints such as dGEMRIC. These results encourage the expanded use of cartilage-sensitive MRI techniques and support further investigations into stratifying OA patients for biologic therapies based on radiographic severity and inflammatory phenotype. Therefore, although KL 4 remains a biologically challenging stage, our data in patients with early to moderate OA grade highlight the potential use of imaging biomarkers, such as dGEMRIC, in monitoring the disease-modifying potential of intra-articular therapies. Incorporating such methods into future studies on KL-4 populations could enhance the utility for identifying responders and filtering patients for selection in clinical studies.

Finally, our findings support a more biologically informed, stratified approach to OA management that incorporates imaging biomarkers alongside PROMs and clinical phenotyping. This study focused on patients with an inflammatory phenotype, which is characterized by joint effusion, stiffness, and signs of synovitis [12]. The selection of an inflammatory phenotype in this trial was based on an increasingly recognized need to align therapeutic strategies with the underlying pathophysiological mechanisms of OA.

The consistent clinical and biochemical improvements observed in this subgroup suggest that inflammatory OA may represent a particularly modifiable phenotype, where intra-articular therapies may exert disease-modifying effects by acting on synovial inflammation. Future studies should directly compare treatment effects across phenotypic subgroups to determine whether therapeutic efficacy varies by underlying pathophysiology [19,21].

Although no statistically significant differences were found in some outcomes, the biological heterogeneity of OA (sex-related, metabolic, biomechanical, and inflammatory factors) may partly explain variability in treatment response. Recent work on OA phenotypes and structural subtypes further underscores that such heterogeneity can mask therapeutic effects in unstratified or small populations [6,10,45,46,47,48]. Our phenotype-based approach aimed to reduce some of this variability, but future larger studies are required to more fully account for these biological differences.

This study has several important strengths. To our knowledge, the present RCT is the first to directly compare non-pharmacological treatments, such as MFAT and HA, in a phenotypically homogeneous cohort of OA patients, alongside validated clinical outcomes and advanced imaging, specifically the dGEMRIC index. The use of a rigorous responder analysis, particularly the implementation of dGEMRIC-based responder thresholds, adds an essential layer of clinical and translational relevance.

The short-to-midterm follow-up of 6 months was deliberately chosen based on the known pharmacokinetics of HA, which rarely demonstrates therapeutic effects beyond 6 to 9 months [49]. In contrast, MFAT has been shown to provide symptom relief for up to two years, which would inherently bias longer follow-up comparisons in favor of MFAT due to its longer-lasting effects [21]. Evaluating both interventions at 6 months thus provides the most clinically meaningful and fair comparison point. Nevertheless, we acknowledge that this timeframe does not allow for conclusions regarding disease-modifying effects, and long-term follow-up studies of 12–24 months will be essential to fully evaluate the durability and structural impact of MFAT treatment.

The use of composite responder definitions based on validated MCID thresholds represents another important methodological strength. By anchoring clinical and imaging improvements to patient-centered thresholds of relevance, such as KOOS Pain ≥ 10, WOMAC Total ≥ 15, and dGEMRIC index ≥ 10%, the study enhances interpretability. It ensures that reported benefits are both statistically and clinically meaningful. Moreover, the integration of both structural (dGEMRIC) and symptomatic (PROMs) endpoints represents a translationally robust approach to outcome assessment. This dual-layered methodology aligns with the evolving paradigm in OA, which emphasizes biologically grounded and patient-centered outcome measures. Simultaneous investigation of changes in cartilage composition and pain reduction increases confidence that the observed improvements correspond to genuine therapeutic effects, rather than isolated placebo or measurement artifacts. This approach reflects an evolution in OA trial design, moving beyond symptom scores toward biologically anchored, patient-relevant outcomes.

Limitations of the present study include the relatively small sample size, which represents a clear constraint on the statistical power and generalizability of our findings. Although strict inclusion and exclusion criteria were applied to achieve phenotypic specificity, this inevitably resulted in a modest final cohort. The predefined 2:1 randomization resulted in a smaller HA group, which may have reduced statistical power for between-group comparisons. This allocation was selected to increase exposure to MFAT and better characterize its effects while retaining HA as an active comparator. Several outcome measures, including changes in dGEMRIC indices and PROMs, showed numerically greater improvements with MFAT but did not reach statistical significance. It is plausible that a larger and more balanced sample size would have amplified these trends and confirmed them as statistically significant, especially in PROM domains where mean differences consistently favored MFAT. To overcome these limitations, future trials should recruit larger patient populations through multicenter and preferably international collaboration, guided by standardized frameworks for OA phenotyping and biologic intervention design.

The study also did not include a placebo arm; instead, HA was selected as the comparator given its role as a recommended standard-of-care injection therapy in knee OA management [9]. A placebo-controlled design was considered inappropriate given the availability of effective standard-of-care treatments and the ethical concerns of withholding therapy from symptomatic patients.

One possible limitation of the single-center design is that it may affect generalizability. This also led to consistent use of inclusion criteria, intervention protocols, and imaging methods.

Another limitation of our study is related to the dGEMRIC segmentation process, which was performed by a single author (a musculoskeletal radiologist with extensive experience). Although such a method could potentially reduce generalizability because no inter-observer variability was assessed, it does significantly increase internal consistency and standardize ROI placement and data interpretation. Single-rater segmentation, although limiting external reproducibility, enhances the reliability of analyses in early-phase trials, where the consistency of ROI placement is crucial for detecting subtle biochemical changes. Subsequent multicenter studies may want to use multiple raters to reassess inter-observer reproducibility; however, this study’s design prioritizes internal validity and the reliability of structural outcome assessment.

Another limitation is that baseline PROMs, such as KOOS Pain, were not used as exclusion criteria to filter out near-maximal baseline scores. This may have led to a ceiling effect in the analysis, potentially underestimating the extent of symptomatic improvement in certain patients.

From a clinical perspective, our findings support the tailored use of intra-articular therapies based on patient phenotype. In patients with severe joint effusion and stiffness, characteristic of inflammatory OA, MFAT pain reduction may be higher and longer-lasting than HA. Although HA is a useful intervention in moderate OA, the limited duration of its effectiveness highlights the need for more durable interventions, especially in patients with active synovitis. Such phenotype-driven approaches may improve treatment efficiency and minimize unnecessary interventions.

With respect to defining dGEMRIC responders, this is the first study to suggest a combined limit of ≥10% in ≥3 out of 7 cartilage regions. Although not yet validated, this threshold provides a pragmatic and reproducible method for early-phase imaging studies, introducing a novel approach for relating imaging changes to individual patient classifications.

By anchoring the dGEMRIC response to a biologically plausible and regionally consistent threshold, this technique eases the way for future standardization and increased consistency. It provides a repeatable framework for responder analysis in imaging-based OA trials. Such an approach should be tested in other populations in further studies and further evaluated to clarify whether it provides the possibility of identifying early “structural responders” who are more likely to have long-term clinical benefit.

The existing correlation between dGEMRIC and KOOS Pain suggests that dGEMRIC could be useful not only as a research tool but as a potential aid in treatment planning and monitoring. This is especially important in the modern era of personalized medicine, where early biologic response could dictate therapeutic escalation, de-escalation, or redirection.

Although MFAT has greater upfront costs than HA, its strong anti-inflammatory properties and long-lasting effects may justify its use in specific patient populations. Cost-effectiveness studies should be conducted to explore whether future decreases in healthcare utilization, pain medication, or surgical conversion rates offset the investment in MFAT. Such analyses are critical before widespread clinical adoption. Cost-effectiveness modeling, which includes not only direct costs but also the value of delayed surgery/medication reduction, will be required to fully define the health–economic value of MFAT.

In addition, our observations in early to moderate knee OA patients provide a basis for extending research to more advanced disease stages, such as KL 4, where cartilage integrity is more significantly damaged. In the future, the use of sensitive imaging biomarkers, such as dGEMRIC, may facilitate the detection of responders in late-stage OA and guide a more individualized treatment strategy. Furthermore, the addition of biomarkers (e.g., cytokines, miRNAs) may further enhance prediction models and improve patient stratification. Complex computational tools, such as machine learning, could offer further insights into response profiles based on pre-treatment structural, biochemical, and clinical features. Such efforts could ultimately pave the way toward precision-guided treatment algorithms for knee OA, integrating clinical, structural, and molecular data.

## 5. Conclusions

This RCT demonstrates that intra-articular injection of MFAT or HA leads to clinically and structurally meaningful improvements in patients with early to moderate, inflammatory knee OA. A significant increase in cartilage glycosaminoglycan (GAG) content was observed in both groups, as assessed by dGEMRIC, indicating favorable changes in cartilage biochemistry following treatment. However, MFAT demonstrated a more pronounced effect on joint-related symptoms, particularly those captured by the KOOS Symptoms subscale, such as stiffness, effusion, and crepitus, suggesting a stronger impact on synovial inflammation and joint homeostasis.

Importantly, dGEMRIC-based structural improvement closely aligned with pain reduction, supporting its validity as a clinically relevant imaging biomarker in OA. The observed concordance between symptomatic and biochemical improvements strengthens the biological plausibility of treatment response, particularly for MFAT.

These findings contribute to the growing evidence base supporting orthobiologics in early OA and underscore the utility of advanced imaging in evaluating disease-modifying potential. While both treatments were well tolerated and clinically beneficial, MFAT may offer superior and more durable benefits in selected patients with inflammatory OA phenotypes. Future multi-center studies with longer follow-up and broader patient inclusion are warranted to define the disease-modifying potential of MFAT and refine patient selection strategies based on phenotype and biomarker response.

## Figures and Tables

**Table 1:** Clinical study inclusion and exclusion criteria. OA—osteoarthritis; BMI—body mass index; CESD-R—Center for Epidemiologic Studies Depression Scale Revised; MRI—Magnetic Resonance Imaging; MOAKS—MRI Osteoarthritis Knee Score; NSAID—nonsteroidal anti-inflammatory Drugs; PT—prothrombin time.

Inclusion Criteria	Exclusion Criteria
Age between 30 and 75 years	Presence of malignant, systemic inflammatory, or other systemic diseases that potentially cause knee pain or systemic inflammation
Kellgren–Lawrence OA grade 2–3 OR Iwano grade 2–3 for patellofemoral OA	BMI > 30 or diagnosed with diabetes
Knee symptoms (pain) for at least 6 months	≥6 tender points distributed above and below the waist, bilaterally and axially
Mechanical axis deviation < 5°	Depression, according to the CESD-R questionnaire
Ability to comply with follow-up and study instructions	Kellgren–Lawrence grade 4 OR Iwano grade 4
Signed informed consent	Kellgren–Lawrence grade 1 AND Iwano grade 1
	MOAKS synovitis/effusion score < 2 (i.e., no effusion on MRI)
	Post-traumatic knee OA
	History of surgical intervention on the affected knee
	Mechanical axis deviation > 5° (varus or valgus)
	Knee instability
	Meniscal or structural lesions as the primary cause of symptoms (e.g., bucket-handle or radial meniscal tears)
	Knee trauma within the last 3 months
	Intra-articular injection within the last 3 months (e.g., corticosteroids, HA, platelet-rich plasma, etc.)
	Other musculoskeletal conditions (e.g., Marfan syndrome, osteogenesis imperfecta) impairing clinical assessment
	Inability to abstain from NSAID use for 7 days before and during the follow-up
	Known allergies to lidocaine or adrenaline
	Coagulopathy, thrombocytopenia, or anticoagulation with PT < 0.70
	Systemic immunosuppressive therapy
	Synovial chondromatosis or pigmented villonodular synovitis
	Active joint infection
	Pregnancy or intention to become pregnant during the study period
	History of chemotherapy or radiotherapy to the limbs or the adipose tissue harvesting site
	Psychiatric disorders impairing compliance
	Anticipated inability to attend follow-up assessments

**Table 2:** Baseline demographic characteristics of study participants. Values are presented as mean ± standard deviation (SD) for continuous variables and percentages for categorical variables. No statistically significant differences were observed between groups, either for age and BMI (Mann–Whitney U test), or for sex distribution (Chi-squared test). MFAT—microfragmented adipose tissue, HA—hyaluronic acid, BMI—body mass index.

	MFAT (n = 35)	HA (n = 18)	*p*-Value
Age (mean ± SD)	53.9 ± 9.0	58.7 ± 9.5	0.104
Sex (% female)	74.3%	77.8%	0.780
BMI (kg/m^2^, mean ± SD)	26.6 ± 2.4	26.8 ± 2.5	0.851

**Table 3:** Descriptive statistics for 6-month changes in KOOS subscales following intra-articular injection of microfragmented adipose tissue (MFAT) or hyaluronic acid (HA). Values represent the mean change from baseline, standard deviation (SD), and median with interquartile range (IQR) for each group, as well as the *p*-value for statistically significant difference between the groups. The Mann–Whitney U test was used for statistical analysis. A statistically significant between-group difference was observed only in the KOOS Symptoms subscale (*p* = 0.008), favoring MFAT. No significant differences were found in other domains (*p* > 0.05). While the Mann–Whitney U test assesses ranked differences, mean values are presented to illustrate the magnitude of clinical improvement. KOOS—Knee Injury and Osteoarthritis Outcome Score; ADL—Activities of Daily Living; Sport/Rec—Sports and Recreational Activities; QoL—Quality of Life.

KOOS Subscore	Group	Mean	SD	Median (IQR)	*p*-Value
ΔKOOS Pain_6M	HA	22.4	22.0	20.8 (34.0)	0.623
	MFAT	23.0	15.0	20.8 (28.5)	
ΔKOOS Symptoms_6M	HA	12.7	16.0	12.5 (18.8)	0.008
	MFAT	25.0	15.6	23.2 (15.2)	
ΔKOOS ADL_6M	HA	19.3	19.6	16.9 (34.9)	0.307
	MFAT	22.6	14.8	25.0 (23.2)	
ΔKOOS Sport/Rec_6M	HA	19.7	23.7	20.0 (35.0)	0.122
	MFAT	29.7	24.6	25.0 (42.5)	
ΔKOOS QoL_6M	HA	23.6	25.4	12.5 (46.9)	0.323
	MFAT	29.8	22.0	31.3 (37.5)	

**Table 4:** Baseline dGEMRIC values and mean percentage change from baseline to 6 months in each anatomical region of interest, stratified by treatment group. All *p*-values are based on the Mann–Whitney U test. MFAT—microfragmented adipose tissue, HA—hyaluronic acid, dGEMRIC—delayed Gadolinium-Enhanced Magnetic Resonance Imaging of the Cartilage.

Region of Interest	MFAT		HA		*p*-Value (MFAT vs. HA)
	Baseline dGEMRIC Mean (ms)	Mean % Change	Baseline dGEMRIC Mean (ms)	Mean % Change	
Lateral femur	499.9	11.3%	519.7	7.7%	0.174
Lateral tibia	487.4	11.5%	507.1	7.5%	0.211
Medial femur	489.1	9.2%	500.7	8.7%	0.737
Medial tibia	440.8	13.3%	428.2	9.2%	0.156
Trochlea	446.2	11.6%	449.9	9.6%	0.574
Lateral patella	452.6	10.7%	464.4	8.2%	0.358
Medial patella	441.0	10.1%	454.8	7.8%	0.343

**Table 5:** Cross-tabulation of dGEMRIC and KOOS Pain responder status (n = 45).

	KOOS Pain Responder	KOOS Pain Non-Responder
dGEMRIC responder	23	0
dGEMRIC non-responder	14	8

## Data Availability

The data presented in this study are available on request from the corresponding author.

## Supplementary Materials

The following supporting information can be downloaded at: https://www.mdpi.com/article/10.3390/biomedicines13092301/s1, Table S1: Descriptive statistics for each KOOS subscale (Pain, Symptoms, Activities of Daily Living [ADL], Sport and Recreation [Sport/Rec], and Quality of Life [QoL]) at baseline, 1 month, and 6 months post-treatment, stratified by treatment group (MFAT and HA).; Table S2: Mean ± standard deviation (SD) values for WOMAC Pain, Stiffness, Function, and Total subscales at baseline, 1 month, and 6 months in the MFAT and HA groups.; Table S3: Descriptive statistics of changes in WOMAC subscale scores (Pain, Stiffness, Function, Total) from baseline to 6 months following intra-articular injection of microfragmented adipose tissue (MFAT) or hyaluronic acid (HA).; Table S4: Mean ± standard deviation (SD) values for VAS scores at rest and during movement at baseline, 1 month, and 6 months in the MFAT and HA groups.; Table S5: Responder status for patient-reported outcome measures (PROMs) based on predefined minimal clinically important difference (MCID) thresholds, following application of ceiling effect exclusion criteria.; Table S6: Mean ± standard deviation (SD) values of dGEMRIC indices at baseline (0M) and at 6 months (6M) in seven cartilage regions of interest (ROI) for both MFAT and HA treatment groups.

## Abbreviations

The following abbreviations are used in this manuscript:

CESD-R	Center for Epidemiologic Studies Depression Scale Revised
dGEMRIC	Delayed Gadolinium-Enhanced Magnetic Resonance Imaging of Cartilage
GAG	Glycosaminoglycan
HA	Hyaluronic acid
IQR	Interquartile range
KOOS	Knee Injury and Osteoarthritis Outcome Score
LF	Lateral femur
LP	Lateral patella
LT	Lateral tibia
MCID	Minimal clinically important difference
MF	Medial femur
MFAT	Microfragmented adipose tissue
MOAKS	MRI Osteoarthritis Knee Score
MP	Medial patella
MRI	Magnetic resonance imaging
MSC	Mesenchymal stem cell
MT	Medial tibia
OA	Osteoarthritis
PPV	Positive predictive value
PROMs	Patient-reported outcome measures
RCT	Randomized controlled trial
ROI	Region of interest
SVF	Stromal vascular fraction
TR	Trochlea
VAS	Visual analogue scale
WOMAC	Western Ontario and McMaster Universities Osteoarthritis Index
