# Supplementary material for: Clinical and dGEMRIC Evaluation of Microfragmented Adipose Tissue Versus Hyaluronic Acid in Inflammatory Phenotype of Knee Osteoarthritis: A Randomized Controlled Trial
Source: Biomedicines. 2025 Sep 19;13(9):2301. doi: 10.3390/biomedicines13092301 (PMC12467587; doi:10.3390/biomedicines13092301)
Supplement: Supplementary file 1 [file biomedicines-13-02301-s001.zip › Supplementary Table S1.pdf]

Supplementary Table S1. Descriptive statistics for each KOOS subscale (Pain, Symptoms, Activities of Daily Living [ADL], Sport and Recreation [Sport/Rec], and Quality of Life [QoL]) at baseline, 1 month, and 6 months post-treatment, stratified by treatment group (MFAT and HA). Values are presented as mean ± standard deviation (SD). Additionally, the table includes within-group comparisons for each subscale across the following time intervals: 1 month vs baseline, 6 months vs baseline, and 6 months vs 1 month. Corresponding p-values were calculated using Wilcoxon signed-rank tests. MFAT – microfragmented adipose tissue; HA – hyaluronic acid.

KOOS subscale	Group	0M (mean ± SD)	1M (mean ± SD)	6M (mean ± SD)	p-value (1M - 0M)	p-value (6M - 0M)	p-value (6M - 1M)
KOOS Pain	MFAT	56.4 ± 19.0	70.8 ± 18.2	79.4 ± 19.2	0.000	0.000	0.000
	HA	57.4 ± 18.7	72.2 ± 16.1	79.8 ± 19.3	0.001	0.001	0.060
KOOS Symptoms	MFAT	62.5 ± 19.8	77.5 ± 16.7	87.5 ± 14.1	0.000	0.000	0.000
	HA	68.8 ± 19.7	80.4 ± 16.0	81.6 ± 21.4	0.001	0.008	0.387
KOOS ADL	MFAT	58.5 ± 19.4	72.4 ± 19.4	81.1 ± 19.6	0.000	0.000	0.000
	HA	59.4 ± 20.5	71.5 ± 17.9	78.7 ± 20.7	0.001	0.002	0.186
KOOS Sport/Rec	MFAT	24.8 ± 22.2	39.6 ± 30.3	54.6 ± 30.7	0.000	0.000	0.000
	HA	25.8 ± 20.6	37.8 ± 22.6	45.6 ± 26.1	0.002	0.004	0.113
KOOS QoL	MFAT	30.0 ± 17.6	43.9 ± 23.4	59.7 ± 22.8	0.000	0.00	0.000
	HA	33.7 ± 18.7	44.8 ± 22.1	57.3 ± 26.7	0.002	0.002	0.028
