# Supplementary material for: Clinical and dGEMRIC Evaluation of Microfragmented Adipose Tissue Versus Hyaluronic Acid in Inflammatory Phenotype of Knee Osteoarthritis: A Randomized Controlled Trial
Source: Biomedicines. 2025 Sep 19;13(9):2301. doi: 10.3390/biomedicines13092301 (PMC12467587; doi:10.3390/biomedicines13092301)
Supplement: Supplementary file 1 [file biomedicines-13-02301-s001.zip › Supplementary Table S2.pdf]

Supplementary Table S2. Mean ± standard deviation (SD) values for WOMAC Pain, Stiffness, Function, and Total subscales at baseline, 1 month, and 6 months in the MFAT and HA groups. p-values reflect within-group comparisons using the Wilcoxon signed-rank test.

WOMAC subscale	Group	0M (mean ± SD)	1M (mean ± SD)	6M (mean ± SD)	p-value (1M - 0M)	p-value (6M - 0M)	p-value (6M - 1M)
WOMAC Pain	MFAT	7.8 ± 3.6	4.7 ± 3.3	3.2 ± 3.8	0.000	0.000	0.001
	HA	7.7 ± 4.0	4.7 ± 3.4	3.4 ± 3.5	0.001	0.002	0.082
WOMAC STIFFNESS	MFAT	3.2 ± 2.1	2.0 ± 1.7	1.2 ± 1.6	0.000	0.000	0.003
	HA	2.7 ± 1.7	1.7 ± 1.5	1.5 ± 1.7	0.004	0.007	0.340
WOMAC FUNCTION	MFAT	28.2 ± 13.2	18.7 ± 13.1	12.8 ± 13.3	0.000	0.000	0.000
	HA	27.7 ± 14.0	19.4 ± 12.2	14.5 ± 14.0	0.001	0.002	0.129
WOMAC TOTAL	MFAT	39.2 ± 17.7	25.4 ± 14.4	17.3 ± 18.3	0.000	0.000	0.000
	HA	38.2 ± 18.8	25.8 ± 16.3	19.4 ± 18.5	0.001	0.001	0.139
