# Supplementary material for: Clinical and dGEMRIC Evaluation of Microfragmented Adipose Tissue Versus Hyaluronic Acid in Inflammatory Phenotype of Knee Osteoarthritis: A Randomized Controlled Trial
Source: Biomedicines. 2025 Sep 19;13(9):2301. doi: 10.3390/biomedicines13092301 (PMC12467587; doi:10.3390/biomedicines13092301)
Supplement: Supplementary file 1 [file biomedicines-13-02301-s001.zip › Supplementary Table S3.pdf]

Supplementary Table S3. Descriptive statistics of changes in WOMAC subscale scores (Pain, Stiffness, Function, Total) from baseline to 6 months following intra-articular injection of microfragmented adipose tissue (MFAT) or hyaluronic acid (HA).

Mean values, median values, standard deviation (SD), interquartile range (IQR), as well as p-values for inter-group statistics using the Mann-Whitney U test are provided for each subscale and treatment group. Negative values indicate symptom reduction and clinical improvement. Including both SD and IQR allows a better understanding of central tendency, dispersion, and potential skewness within patient responses.

WOMAC subscore	Group	Mean	Median	SD	IQR	p-value
ΔWOMAC Pain_6M	HA	-4.3	-4.0	4.3	4.8	0.801
	MFAT	-4.5	-4.0	3.2	5.5	
ΔWOMAC Stiffness_6M	HA	-1.2	-2.0	1.5	2.0	0.174
	MFAT	-2.0	-1.0	1.8	2.0	
ΔWOMAC Function_6M	HA	-13.2	-17.0	13.5	21.0	0.312
	MFAT	-15.4	-11.5	10.0	14.8	
ΔWOMAC Total_6M	HA	-18.8	-24.0	18.5	28.5	0.260
	MFAT	-21.9	-17.0	13.5	20.0	
