# Supplementary material for: Clinical and dGEMRIC Evaluation of Microfragmented Adipose Tissue Versus Hyaluronic Acid in Inflammatory Phenotype of Knee Osteoarthritis: A Randomized Controlled Trial
Source: Biomedicines. 2025 Sep 19;13(9):2301. doi: 10.3390/biomedicines13092301 (PMC12467587; doi:10.3390/biomedicines13092301)
Supplement: Supplementary file 1 [file biomedicines-13-02301-s001.zip › Supplementary Table S4.pdf]

Supplementary Table S4. Mean ± standard deviation (SD) values for VAS scores at rest and during movement at baseline, 1 month, and 6 months in the MFAT and HA groups. p-values reflect within-group comparisons over time using the Wilcoxon signed-rank test.

VAS	Group	0M (mean ± SD)	1M (mean ± SD)	6M (mean ± SD)	p-value (1M - 0M)	p-value (6M - 0M)	p-value (6M - 1M)
VAS resting	MFAT	3.5 ± 2.2	2.4 ± 2.1	1.6 ± 2.3	0.000	0.000	0.001
	HA	3.1 ± 2.3	2.1 ± 1.6	1.3 ± 1.7	0.007	0.003	0.023
VAS movement	MFAT	5.4± 2.2	3.7 ± 2.1	2.8 ± 2.0	0.000	0.000	0.002
	HA	4.8 ± 2.1	3.4 ± 2.5	2.8 ± 2.4	0.003	0.001	0.115
