# Supplementary material for: Clinical and dGEMRIC Evaluation of Microfragmented Adipose Tissue Versus Hyaluronic Acid in Inflammatory Phenotype of Knee Osteoarthritis: A Randomized Controlled Trial
Source: Biomedicines. 2025 Sep 19;13(9):2301. doi: 10.3390/biomedicines13092301 (PMC12467587; doi:10.3390/biomedicines13092301)
Supplement: Supplementary file 1 [file biomedicines-13-02301-s001.zip › Supplementary Table S5.pdf]

Supplementary Table S5. Responder status for patient-reported outcome measures (PROMs) based on predefined minimal clinically important difference (MCID) thresholds, following application of ceiling effect exclusion criteria. KOOS Pain responders were defined as patients with ≥10-point improvement from baseline to 6 months and baseline scores <85. VAS Movement responders required >2-point improvement and baseline ≥ 3. WOMAC Total responders required ≥15-point improvement and baseline >15. The table presents the number and percentage of responders and non-responders within each group (MFAT and HA), as well as the number of participants excluded per outcome due to ceiling effects. All responder proportions are based on patients remaining after exclusion. MFAT – microfragmented adipose tissue; HA – hyaluronic acid.

PROM	ceiling effect cut off value	MCID	number of patients after ceiling exclusion	GROUP	Number of responders	Number of non-responders	% of Responders
KOOS Pain	≥85	10	41	HA	12	4	75.0%
				MFAT	20	5	80.0%
VAS movement	≤2	3	44	HA	7	8	46.7%
				MFAT	17	12	58.6%
WOMAC total	≤15	15	48	HA	10	4	62.5%
				MFAT	22	10	68.8%
