# Supplementary material for: Clinical and dGEMRIC Evaluation of Microfragmented Adipose Tissue Versus Hyaluronic Acid in Inflammatory Phenotype of Knee Osteoarthritis: A Randomized Controlled Trial
Source: Biomedicines. 2025 Sep 19;13(9):2301. doi: 10.3390/biomedicines13092301 (PMC12467587; doi:10.3390/biomedicines13092301)
Supplement: Supplementary file 1 [file biomedicines-13-02301-s001.zip › Supplementary Table S6.pdf]

Supplementary Table S6. Mean ± standard deviation (SD) values of dGEMRIC indices at baseline (0M) and at 6 months (6M) in seven cartilage regions of interest (ROI) for both MFAT and HA treatment groups. p-values reflect within-group comparisons: paired t-test was used for all MFAT ROIs except for the medial tibia (Wilcoxon signed-rank test), while all HA comparisons were analyzed using the Wilcoxon signed-rank test. MFAT – microfragmented adipose tissue; HA – hyaluronic acid.

	MFAT			HA		
**ROI**	**0M (mean ± SD)**	**6M (mean ± SD)**	**p- value**	**0M (mean ± SD)**	**6M (mean ± SD)**	**p- value**
**lateral femur**	499.9 ± 60.3	555.0 ± 65.5	0.000	519.7 ± 55.4	560.1 ± 76.9	0.001
**lateral tibia**	487.4 ± 77.3	541.6 ± 80.9	0.000	507.1 ± 92.0	543.8 ± 97.1	0.006
**medial femur**	489.1 ± 87.1	532.7 ± 96.7	0.000	500.7 ± 68.0	543.5 ± 80.7	0.002
**medial tibia**	440.9 ± 79.8	497.6 ± 89.7	0.000	428.2 ± 78.0	464.4 ± 73.0	0.002
**trochlea**	446.2 ± 65.9	497.5 ± 81.5	0.000	449.9 ± 61.0	494.9 ± 88.5	0.000
**lateral patella**	438.9 ± 114.0	485.3 ± 128.9	0.000	412.8 ± 164.2	446.4 ± 178.7	0.001
**medial patella**	441.0 ± 79.0	485.5 ± 91.2	0.000	404.2 ± 162.4	435.6 ± 176.2	0.001
